# Study on Anisotropic Mechanical Properties of Single-Crystal Silicon at Different Strain Rates

**DOI:** 10.3390/mi16070744

**Published:** 2025-06-25

**Authors:** Zhongwang Tian, Wei Xue, Wenzhong Lou, Min Liu, Hengzhen Feng, Xiaoxia Wang, Shiteng Li, Shaokuan Wu

**Affiliations:** 1 School of Mechatronical Engineering, Beijing Institute of Technology, Beijing 100081, China; tianzw1205@163.com (Z.T.); fenglei@bit.edu.cn (H.F.); 18729865852@163.com (S.L.); 2 Xi’an Institute of Electromechanical Information Technaology, Xi’an 710065, China; Nuc_xuewei@163.com (W.X.); minl381@163.com (M.L.); wxx_1204@163.com (X.W.); 3 Institute of Functional Nano & Soft Materials (FUNSOM), Soochow University, Suzhou 215123, China; wskstrive@163.com

**Keywords:** single-crystal silicon, crystallographic orientation, nanoindentation, mechanical properties, tension–compression

## Abstract

To examine the impact of the strain rate on the anisotropic mechanical characteristics of single-crystal silicon, nanoindentation and micro-tensile–compression tests were performed. This study analyzed the effects of varying crystal orientations at different strain rates on load–displacement behavior, elastic modulus, hardness, fracture toughness, and true stress–strain responses. The nanoindentation results showed that at room temperature, single-crystal silicon exhibited an elastic recovery rate of approximately 42%. Notably, the elastic modulus remained unaffected by strain rate variations, whereas hardness increased with higher strain rates. Fracture toughness at room temperature displayed marked anisotropy, with the <100> orientation exhibiting the lowest value at 0.691 MPa·m^1/2^ and the <110> orientation showing the highest one at 0.797 MPa·m^1/2^. Additionally, tensile and compression experiments revealed that the fracture strength of <100>-oriented silicon increased from 117 MPa at a strain rate of 0.001 s^−1^ to 550 MPa at a strain rate of 0.01 s^−1^.

## 1. Introduction

Single-crystal silicon exhibits superior resistance to high temperatures and corrosion compared with metals and polymers, boasting mature technology, low costs, and ease of processing. These attributes render it suitable as a substrate material for microelectromechanical systems (MEMSs) and nanoelectromechanical systems (NEMSs) [1,2,3,4]. Single-crystal silicon substrates find extensive application in military and civilian equipment, such as artillery and automobiles, in devices like pressure sensors, inertial switches, and gyroscopes [5,6]. Silicon microstructures—including cantilevers and other sensitive components—constitute fundamental elements of MEMS devices. At micrometer scales, these structures exhibit pronounced size effects [7,8,9], wherein their mechanical properties (elastic modulus, fracture toughness, hardness, residual stress, and fatigue behavior) significantly influence crack initiation, propagation, and ultimately, the reliability of microstructural fracture.

Extensive research has been dedicated to elucidating the mechanical properties of monocrystalline silicon globally. Rickhey et al. investigated crystal plasticity under varied loads across different crystallographic orientations [10,11,12,13,14], while Masolin et al. explored the anisotropy in size effects and property variations [15,16,17,18,19]. Despite advancements, the mechanisms governing microstructural damage in real-world engineering contexts remain elusive, alongside the strain rate sensitivity of mechanical responses. To bridge these gaps, this study employs nanoindentation and micro-tensile testing to systematically assess how the surface quality and mechanical properties of monocrystalline silicon vary with crystal orientation under diverse strain rates. A comprehensive analysis is presented on the anisotropic surface property alterations concerning the principal crystal planes: <100>, <110>, and <111>. This work furnishes a theoretical and empirical foundation for evaluating how mechanical processing at micro–nanometer scales affects the fidelity of fracture damage in complex systems.

## 2. Nanoindentation Testing Technology and Sample Pretreatment

### 2.1. Nanoindentation Testing

Nanoindentation testing is the prevailing technique for characterizing the mechanical properties of MEMS materials. Through the analysis of the load–displacement curves generated by an indenter on a specimen, key parameters, such as hardness, elastic modulus, and elastic recovery rate, are derived. This study employs the Oliver–Pharr method, a widely adopted nanoindentation theory that relies on contact depth and stiffness measurements [20,21,22]. Experiments were conducted using a Nano Indenter G200 equipped (KLA Corporation, Milpitas, CA, USA) with a Berkovich three-sided pyramidal diamond tip. The indenter’s properties include a Poisson’s ratio of 0.07, an elastic modulus of 1141 GPa, face angles of 65.03° and 77.05°, a base edge-to-depth ratio of 7.5315, and a tip radius of 20 mm. For single-crystal silicon samples, the Poisson’s ratio was set to 0.22, with a maximum indentation depth limited to 1200 nm to ensure accuracy.

The elastic modulus of the material under examination can be determined using the following formula:

(1) $$\frac{1}{E_{r}}=\frac{1-\nu^{2}}{E}+\frac{1-{\nu_{i}}^{2}}{E_{i}}.$$

The reduced modulus, *E_r_*, in the equation encapsulates the synergistic elastic response of both the specimen and the indenter. Here, *E_i_* and ν*_i_* denote the elastic modulus and Poisson’s ratio of the indenter, while E and u correspond to those of the specimen material.

The hardness of the material under examination can be determined by applying the fundamental definition of hardness:

(2) $$H=P_{\max}/A_{c}.$$

The formula defines *P*_max_ as the maximum indentation load (in millinewtons, mN) and *A_c_* as the contact area of the indenter. Specifically, for a Berkovich indenter, hchc represents the contact depth.

The indentation fracture toughness of single-crystal silicon was tested and analyzed using the Lawn–Evans–Marshall (LEM) elastic–plastic indentation fracture model [23,24,25]. Lawn and colleagues differentiated the effects of indentation into irreversible plastic deformation and reversible elastic deformation regions. The irreversible plastic region promotes radial crack propagation, whereas the reversible elastic region inhibits it. They established a method for characterizing fracture toughness: for semi-penny-shaped cracks with sufficient radial crack extension, the distance *c* from the crack tip to the indentation center point is measured. The fracture toughness *K_IC_* can then be determined using Equation (3):

(3) $$K_{IC}=\delta {\left(\frac{E}{H}\right)}^{1/2}\frac{F}{c^{3/2}}.$$

The fracture toughness of the specimen material is denoted by *K_IC_*, while *E* represents its elastic modulus, and *H* signifies hardness. The parameter *c* measures the distance from the crack tip to the indentation center, with *δ* serving as a determinable coefficient. The indentation load is given by *F* (in millinewtons, mN). For scenarios where radial cracks attain full extension under a Brinell indenter, the value of *δ* is established at 0.016.

### 2.2. Tensile and Compression Testing

Nanoindentation tests can determine the relationships between load, displacement, and material properties such as elastic modulus and hardness at low strain rates for single-crystal silicon. They also provide valuable insights into fracture mechanics. However, these tests cannot directly give the true stress–strain relationship of materials under engineering conditions. In contrast, uniaxial tensile and compressive tests can measure the stress–strain curves of materials during mechanical testing, with material stiffness and strength typically reflected in changes in the stress–strain relationship [26,27]. This paper employs a ZQ-950 series universal tensile and compression testing machine to conduct tensile and compressive tests on single-crystal silicon, aiming to assess the mechanical properties of microscale-structured materials.

### 2.3. Test Samples

**Figure 1:**
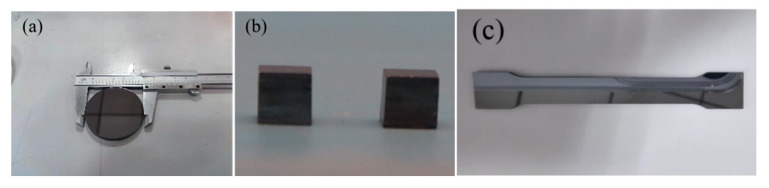
Single-crystal silicon test sample. (**a**) Single-crystal silicon wafer. (**b**) Two 5 × 5 × 5 mm^3^ cubic samples. (**c**) “Dog-bone” shaped tensile specimen.

Figure 1 illustrates the single-crystal silicon specimens employed in diverse experiments. Four-inch wafers were precision-cut into designated shapes and dimensions for testing via a diamond wire saw. Nanoindentation and compression test samples assumed cubic morphology, measuring 5 × 5 × 5 mm^3^. Tensile test specimens, characterized by a <100> crystallographic orientation, featured a “dog bone” design with an overall length of 49 mm, a gauge length of 20 mm, and a thickness of 200 μm.

## 3. Effects of Different Strain Rates on Mechanical Properties of Single-Crystal Silicon

Indentation mechanical tests were performed on single-crystal silicon with varying crystal orientations at six distinct strain rates. The impact of these strain rates on the elastic modulus, hardness, and fracture toughness was systematically examined. The selected strain rates were 0.01 s^−1^, 0.05 s^−1^, 0.10 s^−1^, 0.25 s^−1^, 0.50 s^−1^, and 1.00 s^−1^. At room temperature, the loading procedure for each strain rate was replicated five times (yielding a total of 30 data points), and the continuous stiffness measurement technique was employed to determine the samples’ elastic modulus and hardness.

### 3.1. Load–Displacement Curve

**Figure 2:**
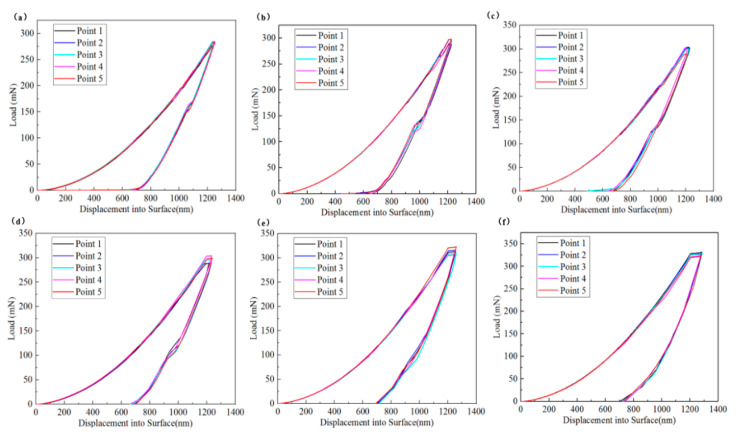
Load–displacement curves of single-crystal silicon in <100> direction at different strain rates: (**a**) 0.01 s^−1^; (**b**) 0.05 s^−1^; (**c**) 0.1 s^−1^; (**d**) 0.25 s^−1^; (**e**) 0.5 s^−1^; (**f**) 1 s^−1^.

Figure 2 illustrates the load–displacement curves of single-crystal silicon in the <100> direction at varying strain rates. The observations reveal that at identical strain rates, the curves for each test point closely overlap, evidencing the repeatability and reliability of the experimental data within each group. Notably, the residual indentation depth (h_f_) is consistently measured at around 700 nm, while the maximum indentation depth (h_max_) and load (P_max_) are approximately 1200 nm and 330 mN, respectively. The indentation curves for single-crystal silicon with orientations of <110> and <111> demonstrate repeatability and reliability at various strain rates. During the loading stage, the depth of the indenter penetrating into the specimen increases with the load. Conversely, during the unloading stage, the penetration depth decreases as the load is reduced, indicating the elastic recovery of single-crystal silicon. The elastic depth is approximately 500 nm. However, after unloading, the indenter does not return to the initial indentation surface but leaves a partially irrecoverable residual indentation depth. As the indentation load increases, both the maximum indentation depth and the residual indentation depth also increase, indicating that irreversible plastic deformation occurs during the indentation process. In the unloading segment of the curve, noticeable pop-out and elbow phenomena can be observed, suggesting that phase transformation occurs in single-crystal silicon during the indentation test.

**Figure 3:**
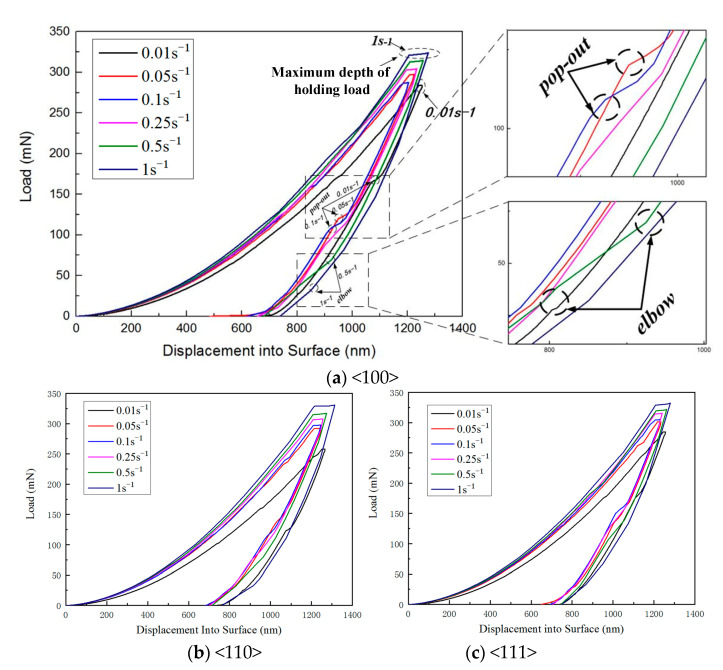
The study examines load–displacement indentation curves of single-crystal silicon for varying strain rates and crystallographic orientations.

The average value of the test data at various testing points under the same crystal orientation was analyzed to examine the load–displacement indentation curves of single-crystal silicon for different strain rates and crystal orientations, as illustrated in Figure 3. As shown in Figure 3a, for the same crystal orientation, an increase in the strain rate results in a gradual increase in load depth. Additionally, the holding depth at higher strain rates is greater than that at lower strain rates during the holding phase. Consequently, the direction of lateral displacement at the maximum load position is smoother at higher strain rates, whereas it is more abrupt at lower strain rates. The shape of the unloading segment of the single-crystal silicon indentation curve varies with strain rate. Some curves exhibit a clear pop-out step, while others display a significant elbow phenomenon during the unloading stage, as depicted in Figure 3a. At lower strain rates (0.01 s^−1^, 0.05 s^−1^, and 0.1 s^−1^), pop-out phenomena are more prevalent during unloading. In contrast, at higher strain rates (0.25 s^−1^, 0.5 s^−1^, and 1 s^−1^), elbow phenomena are more common. This indicates that the transition phase after unloading in single-crystal silicon indentation is strain rate-dependent. Specifically, the curves tend to exhibit elbow phenomena at faster strain rates and pop-out phenomena at slower strain rates. The magnitude of the strain rate is positively correlated with the unloading rate, consistent with the findings obtained by Domnich et al. [28,29], who observed that elbow phenomena occur at faster unloading rates and pop-out phenomena at slower unloading rates. Pop-out phenomena typically occur in the middle-to-early stages of the unloading phase, while elbow phenomena generally occur in the later stages.

The morphology of the unloading portion in single-crystal silicon indentation curves, characterized by features like pop-out and elbow, is intimately tied to the kinetics of phase transformations. Research indicates that the emergence of a pop-out typically signifies a transition from a high-pressure silicon phase (e.g., Si-III or Si-XII) back to a less dense phase upon unloading. Conversely, the observation of an elbow might denote either elastic rebound following swift unloading or the alleviation of locally confined residual stresses [30,31]. At low strain rates (0.01 s^−1^ to 0.1 s^−1^), the slower unloading rate permits extended time for phase transformation completion, leading to a displacement surge during the early-to-middle unloading stages, manifesting as a pop-out step. This temporal allowance facilitates volume expansion associated with phase changes. Conversely, at high strain rates (0.25 s^−1^ to 1 s^−1^), rapid unloading suppresses full phase transition, predominantly inducing elastic recovery or partial transformation, which results in a late-stage unloading curve bend (“elbow”). The observed strain rate dependence implies that phase transformation criticality, such as energy barriers, is influenced by unloading velocity, with accelerated rates impeding the structural relaxation necessary for transformation. The experimental data further indicate that the pop-out initiation load (P-po) scales with maximum indentation load (P-max) yet maintains a consistent P-po/P-max ratio between 0.2 and 0.5, suggesting that phase transformation onset depends not solely on the strain rate but also on local stress conditions and energy buildup. The prevalence of the elbow feature at high rates may be link with elastic energy rapid release or microcrack dynamics. Thus, the sensitivity of single-crystal silicon’s unloading behavior to the strain rate underscores its intricate phase transformation dynamics: slow rates enable time-sufficient transformations causing pop-outs, whereas fast rates favor elastic rebound or nonequilibrium processes forming elbows. These findings align with Domnich et al.’s studies on unloading rate effects, reinforcing the inherent connection between the strain rate and phase-change responses.

The pop-out and elbow phenomena observed in the indentation curves arise from the intense local strain and stress generated beneath the indenter within the material during testing [32,33,34,35]. These elevated stresses induce plastic deformation through dislocation activity and trigger phase transformations from crystalline phases to denser crystalline or amorphous forms under pressure. In single-crystal silicon, for instance, the material transitions from Si-I to Si-II during loading. Upon unloading, metastable Si-II reverts to either amorphous silicon (α-Si), Si-XII, or Si-III, depending on factors such as the unloading rate and specific conditions. The transient transformation of Si-II to the less dense Si-XII and Si-III leads to a sudden increase in material volume beneath the indenter, causing a step-like pop-out phenomenon. Conversely, the elbow phenomenon occurs because Si-II does not have sufficient time to transform into Si-XII and Si-III at faster unloading rates, instead transitioning more slowly into α-Si.

**Figure 4:**
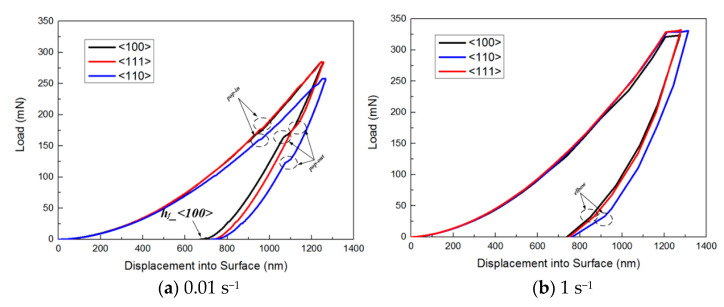
Load–displacement indentation curves were obtained for single-crystal silicon at a consistent strain rate but for varying crystal orientations.

The data analysis of the nanoindentation test results for three different crystallographic orientations of single-crystal silicon at the highest strain rate (1 s^−1^) and the lowest strain rate (0.01 s^−1^) is presented in Figure 4 and Table 1. As shown in Figure 4a, at various strain rates, the <100>-, <111>-, and <110>-oriented samples exhibit smooth and continuous nonlinear loading curves during the initial loading phase. However, as the penetration depth increases, a pop-in phenomenon occurs in the later stages of loading. This pop-in event is likely associated with phase transformation or brittle fracture behavior. During indentation, if the specimen’s surface fractures, the indenter penetrates more deeply due to the sudden loss of material support, resulting in an abrupt increase in penetration depth (pop-in). This is typically accompanied by a sudden load decrease, indicating that single-crystal silicon undergoes brittle fracture under high loads.

In the unloading phase, all three crystallographic orientations exhibit a pop-out phenomenon. Notably, the <111> orientation shows the pop-out earlier, followed by the <100> orientation, with the <110> orientation last. This sequence suggests that at low strain rates, the <110>-oriented specimen undergoes phase transformation later than the others. Additionally, the residual depth for the <100> orientation is smaller, indicating greater elasticity. The degree of the pop-out phenomenon during unloading also varies: the <111> orientation exhibits a significantly gentler step compared with the <110> and <100> orientations. This observation suggests that the <111> orientation undergoes a more gradual phase transformation process involving plastic deformation during the transition from metastable Si-II to other phases.

Figure 4b illustrates that at high strain rates, the loading, holding, and unloading curves for specimens with <100>, <111>, and <110> crystal orientations exhibit remarkable smoothness, devoid of abrupt jumps or step-like features. Notably, during the holding phase, the prolonged depth maintenance at high strain rates compared with low ones facilitates a seamless transition at the peak load. Conversely, the conclusion of the unloading stage is marked by an “elbow” phenomenon, a consequence of the indenter’s accelerated withdrawal rate. As shown in Table 1, the elastic indentation depth of single-crystal silicon is approximately 500–600 nm, with an elastic recovery rate of about 42%. This value is consistent with the results of Panich et al.’s tests, which indicate that the elastic recovery rate of nanoindentation for monocrystalline silicon at room temperature typically ranges from 20% to 50% [36]. The crystal orientation and strain rate do not affect these measurements.

### 3.2. Elastic Modulus and Hardness

**Figure 5:**
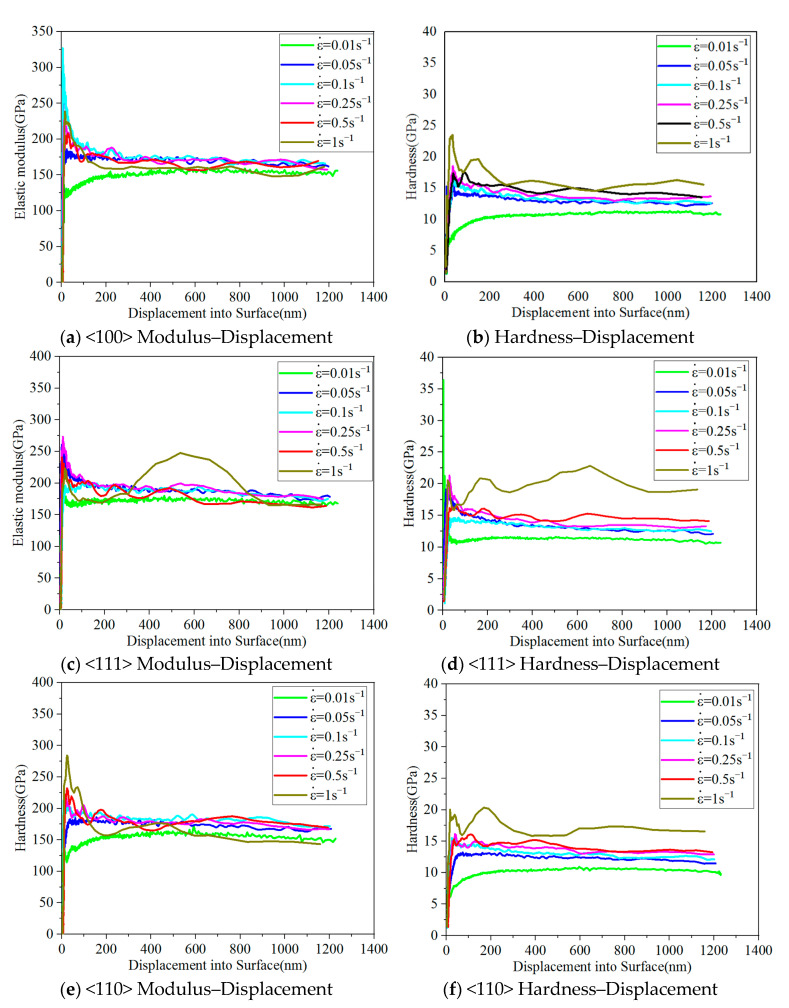
Elastic modulus and hardness curves of single-crystal silicon for varying crystal orientations and strain rates.

Figure 5 illustrates the elastic modulus and hardness curves of single-crystal silicon in the <100>, <111>, and <110> directions at varying strain rates. Initially, both the elastic modulus and hardness exhibit significant fluctuations as the indentation depth increases. However, once the indentation depth reaches approximately 600 nm, these values stabilize. Notably, the elastic modulus remains unaffected by changes in the strain rate, whereas the hardness increases considerably.

**Figure 6:**
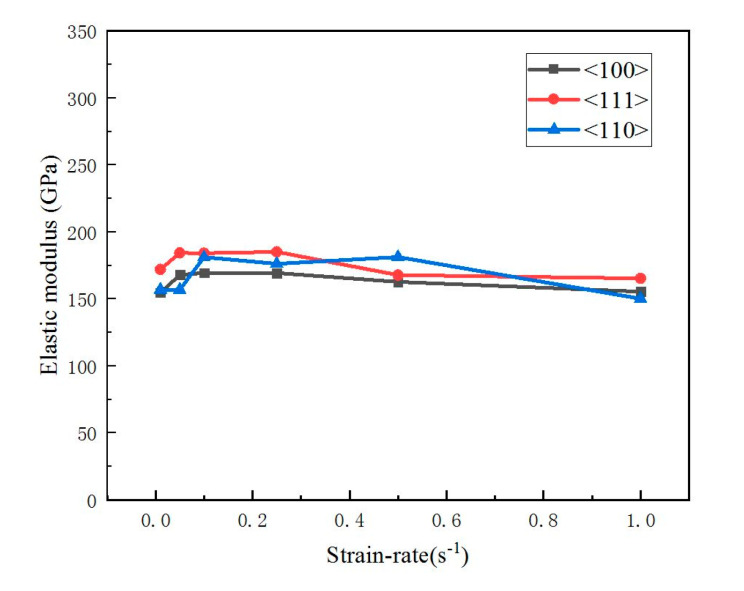
This study investigates the correlation between the elastic modulus of single-crystal silicon and its crystallographic orientation at varying strain rates.

**Figure 7:**
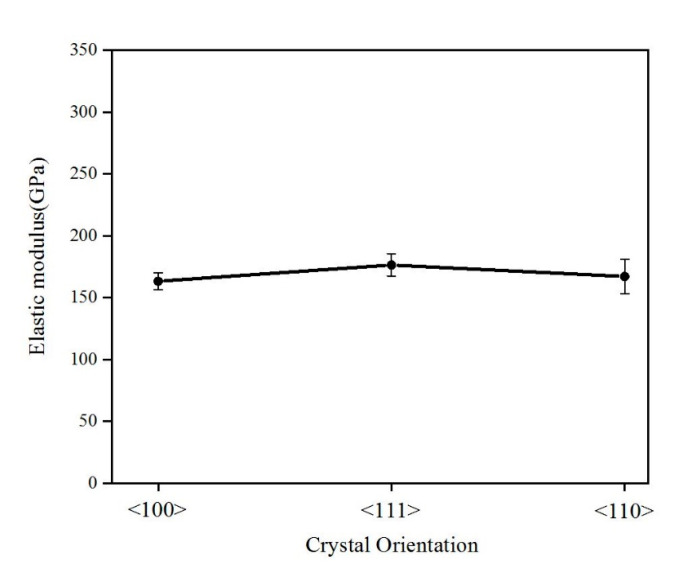
Average elastic modulus of single-crystal silicon in different crystallographic directions.

**Figure 8:**
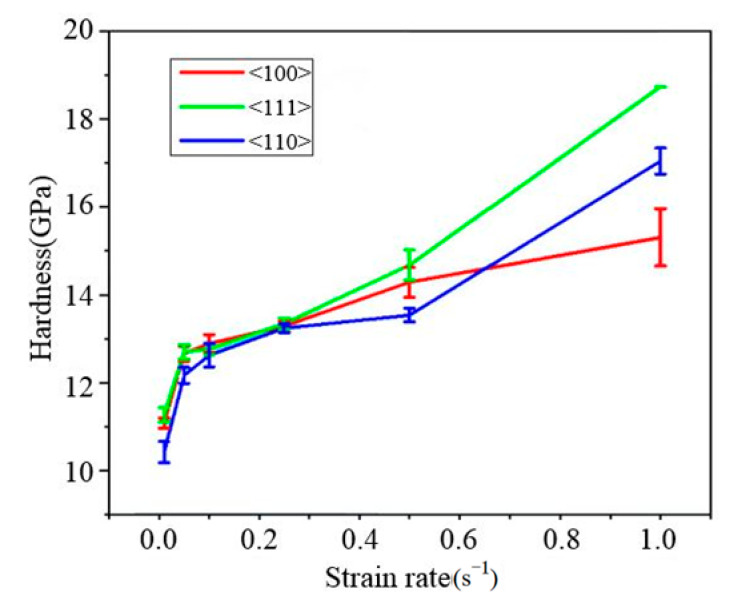
The relationship between the hardness and the strain rate of single-crystal silicon in different crystal orientations.

The elastic modulus and hardness values of single-crystal silicon in the <100>, <111>, and <110> orientations were determined by averaging data between the indentation depths of 600 nm and 1100 nm, as presented in Table 2. Figure 6 demonstrates the strain rate effect on the elastic modulus across different crystal orientations. Notably, the elastic modulus of single-crystal silicon remains relatively constant across varying strain rates, validating its assumption as a constant in engineering applications. Figure 7 compares the average elastic moduli for the three orientations, <100> (163.34 GPa), <111> (176.53 GPa), and <110> (167.18 GPa), with the <111> orientation exhibiting marginally higher values. Figure 8 illustrates the strain rate dependence of hardness for each orientation. Hardness increases with the strain rate; for example, as the strain rate rises from 0.01 s^−1^ to 1 s^−1^, the hardness of <100>, <111>, and <110> silicon increases from 11.08 GPa to 15.31 GPa, from 11.27 GPa to 18.73 GPa, and from 10.43 GPa to 17.04 GPa, respectively. Consequently, <111>-oriented silicon displays significantly greater hardness compared with the other orientations.

**Figure 9:**
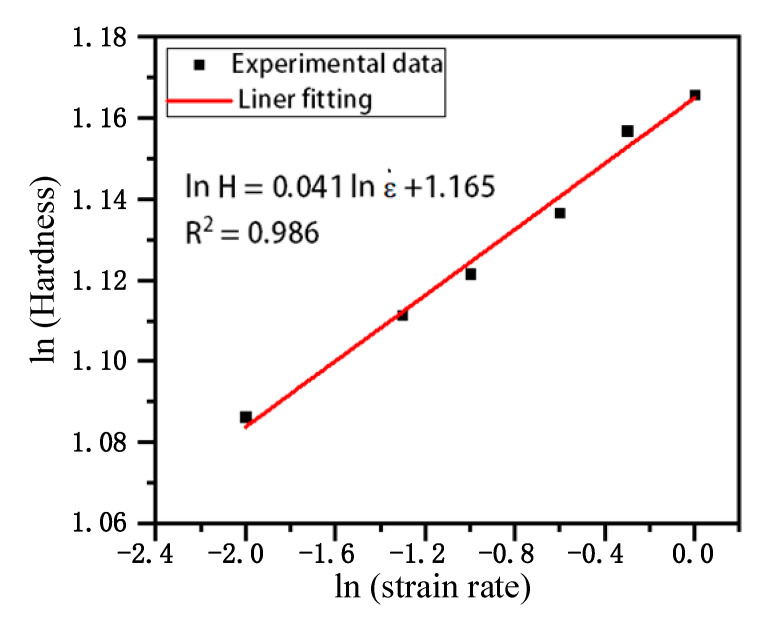
Hardness and strain rate logarithmic function for <100> crystallographic orientation single-crystal silicon.

As the strain rate increases, the region of plastic deformation beneath the indenter expands, resulting in elevated flow stress. This heightened deformation resistance within the confined contact area further contributes to an increase in material hardness, a phenomenon known as strain hardening. To quantify the strain rate sensitivity of monocrystalline silicon, the strain rate sensitivity index mm was introduced and expressed through a natural logarithmic function, capturing the impact of the strain rate on hardness [14,37]. Consequently, the relationship between hardness and the natural logarithm of the strain rate was employed for quantitative analysis, with the fitting formula illustrated in Figure 9. A correlation coefficient of 0.9 for the <100> crystallographic orientation underscores the validity and reliability of the findings.

### 3.3. Study on Anisotropy of Fracture Toughness in Monocrystalline Silicon

Single-crystal silicon exhibits significant brittleness at room temperature, rendering it susceptible to surface damage or even fracturing under contact loading. Given its inherent brittleness, the fracture response of single-crystal silicon varies with different crystallographic orientations when subjected to contact loads. To precisely characterize this behavior, scanning electron microscopy (SEM) was employed to examine the residual surface morphologies post-indentation tests, conducted across a strain rate spectrum of 0.01 s^−1^ to 1.00 s^−1^.

(a) Relationship between crack propagation and different strain rates

**Figure 10:**
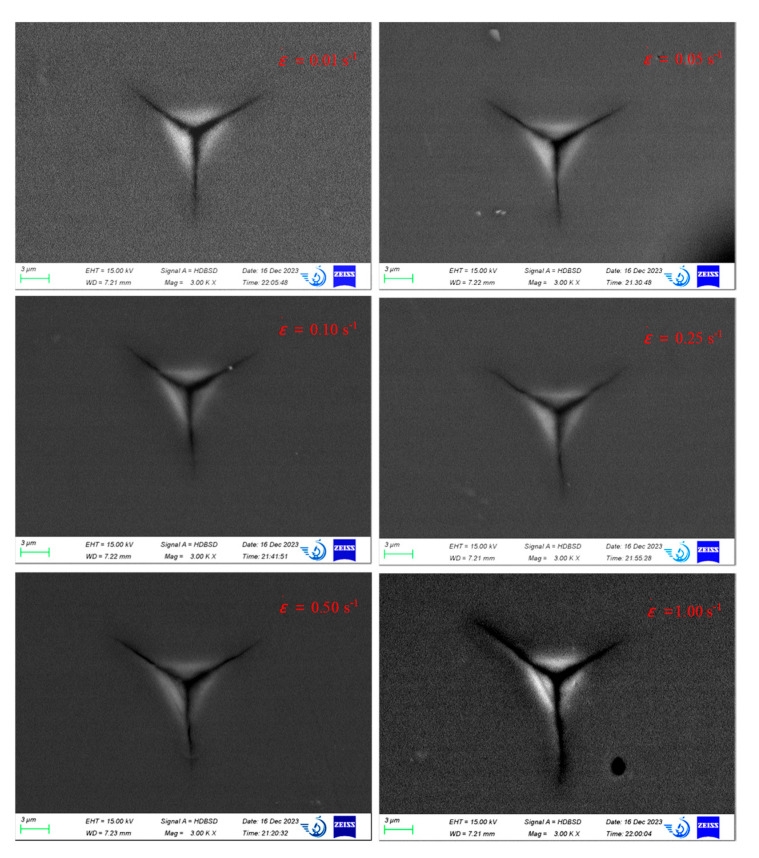
Indentation morphology of single-crystal silicon in <100> crystal orientation at different strain rates.

Figure 10 illustrates the residual indentation morphology of single-crystal silicon along the <100> crystal orientation at varying strain rates. During testing, the indentation depth progressively increases until the applied load reaches a maximum of 300 mN. The resulting indentation morphology closely resembles that observed at a constant loading rate with the same maximum load. As the strain rate increases, the residual indentation area of the single-crystal silicon expands. Notably, radial cracks surrounding the indentation pits are evident at all strain rates, with both the length and width of these cracks increasing as the strain rate rises. This observation suggests a pronounced strain rate size effect in the experimental phenomena.

From the perspective of radial crack propagation, at strain rates ranging from 0.01 s^−1^ to 0.1 s^−1^, the residual morphology of the sample indicates that radial crack extension is primarily concentrated around the vertices of the triangular indenter. When the strain rate reaches 0.25 s^−1^, a noticeable extension of cracks occurs at the vertices, with the length, width, and depth of the extensions increasing as the strain rate increases. At a strain rate of 1 s^−1^, the radial cracks extend significantly beyond the indentation area, resulting in permanent damage. Thus, it can be concluded that crack propagation initiates at a strain rate of approximately 0.25 s^−1^.

(b) Fracture toughness

Fracture toughness quantifies a material’s resistance to crack propagation and is typically characterized by the critical stress intensity factor, *K_IC_*, measured in units of MPa⋅m^1/2^. Investigating *K_IC_* is crucial to evaluating the mechanical properties of engineering materials and determining the microstructural parameters of brittle materials [38,39]. To determine the fracture toughness of single-crystal silicon across various crystal orientations, radial crack lengths in the indentation micro-region were measured, and *K_IC_* was calculated. The Lawn–Evans–Marshall elastic–plastic indentation fracture model, which requires fully extended radial cracks, necessitated indentations under a 300 mN load. The crack lengths at three corners of each indentation were measured using ImageJ 1.8.0 software. For reliability, the average of five indentation tests per orientation was reported, as summarized in Table 3, with the <100> orientation exhibiting the longest radial cracks.

**Figure 11:**
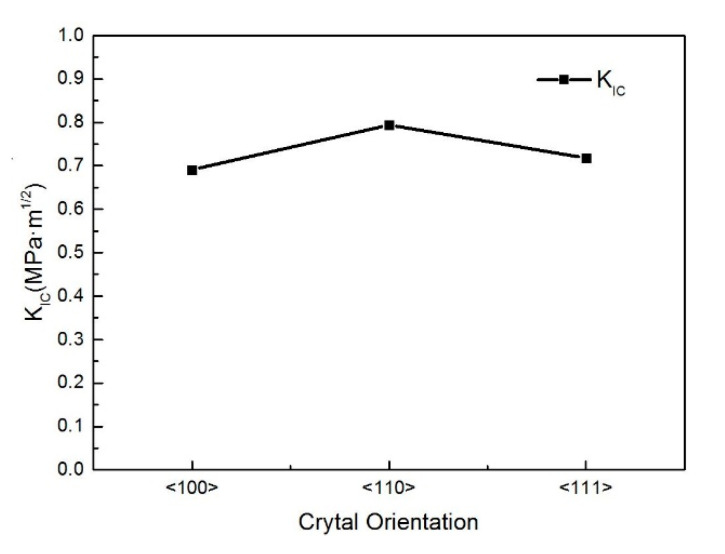
Analysis results of fracture toughness in different crystallographic orientations of single-crystal silicon.

Figure 11 and Table 4 reveal that at room temperature, the fracture toughness of single-crystal silicon exhibits pronounced orientation dependence. The toughness values decrease in the sequence <110> > <111> > <100>. Notably, the <100> orientation has the lowest fracture toughness (0.691 MPa·m^1^/^2^), whereas the <110> orientation displays the highest value (0.797 MPa·m^1^/^2^), representing a 14.9% increase over the <100> orientation. The experimental results obtained by Gallo P et al. indicate that the fracture toughness of monocrystalline silicon does not exhibit significant differences across different crystal orientations, with a fracture toughness value of approximately 1 MPa·m^1/2^ [40]. This finding aligns with the reported fracture toughness range of 0.75–1.08 MPa·m^1/2^ from both this study and macroscale tests. Notably, the value is independent of crystal orientation and specimen size. Consequently, when processing or engineering single-crystal silicon, its inherent anisotropy must be taken into account. Specifically, silicon in the <110> orientation exhibits superior processability and reduced susceptibility to brittle fracture, leading to enhanced surface quality post-processing.

Table 5 and Table 6 present the fracture toughness analysis results for two samples with a <100> crystal orientation at varying strain rates. The data reveal that while both the maximum load and crack length increase with the strain rate, the fracture toughness decreases. This suggests that as loading escalates, cracks propagate fully, leading to a substantial reduction in fracture toughness around dimple sites. Consequently, the material becomes more prone to brittle fracture at higher strain rates.

**Figure 12:**
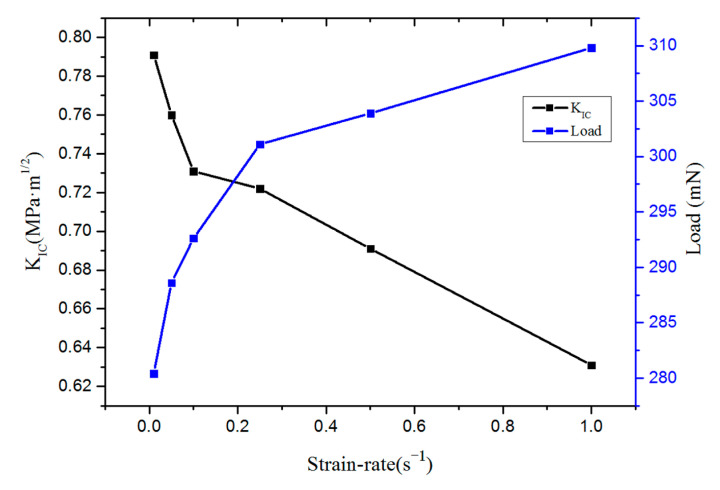
The average fracture toughness of <100> single-crystal silicon at different strain rates.

The average fracture toughness values for two samples at various strain rates are presented in Table 7 and illustrated in Figure 12. It can be concluded that the fracture toughness of monocrystalline silicon in the <100> crystal orientation is inversely proportional to the loading rate across different strain rates. High stress, which results in high strain rates, is a critical factor in resisting crack initiation and propagation. The fracture behavior of the <110> and <111> crystal orientations mirrors that of the <100> orientation.

## 4. Real Stress–Strain Relationship of Single-Crystal Silicon at Different Strain Rates

**Figure 13:**
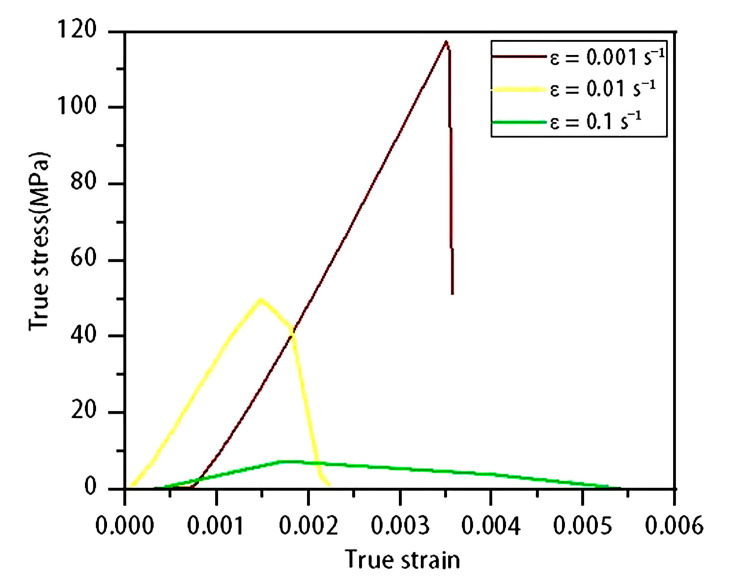
True stress–strain curves at different strain rates.

To determine the true stress–strain relationship of single-crystal silicon at various strain rates, tensile–compression tests were conducted at low strain rates by using a tensile testing machine. The selected strain rates for the tests were 0.001 s^−1^, 0.01 s^−1^, and 0.1 s^−1^. Due to the specimens’ brittle nature, small size, and thinness, several challenges were encountered: (1) specimens were prone to damage during handling; (2) clamping the ends directly with wedge-shaped fixtures caused cracking; (3) attaching metal strips to the specimens often resulted in breakage due to the mismatch in weight and fragility; and (4) excessive clamping force could fracture the specimens in the middle. After multiple tensile testing attempts, the true stress–strain curves obtained are shown in Figure 13. The curve for the strain rate of 0.001 s^−1^~0.001 s^−1^ exhibits brittle fracture around 117 MPa, characterized by constant strain and an instantaneous stress drop, indicating a valid test result. In contrast, the curves for the strain rates of 0.01 s^−1^ and 0.1 s^−1^ deviate significantly, likely due to debonding during stretching, causing fractures outside the standard gauge length and failing to meet the expected experimental outcomes.

**Figure 14:**
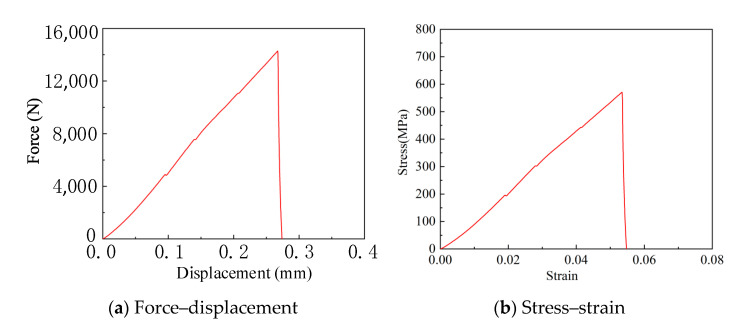
The true stress–strain relationship at a strain rate of 0.01 s^−1^.

The failure observed in tensile tests at the strain rates of 0.01 s^−1^ and 0.1 s^−1^ prompted the use of compression tests for specimens at the strain rate of 0.01 s^−1^. The test samples were 5 × 5 × 5 mm^3^ <100>-oriented single-crystal silicon cubes. The results are presented in Figure 14. As the applied pressure increased, so did the compressive displacement of the sample, indicating that the stress experienced by the sample rose with the increase in true strain. The sample fractured under a compressive load of 14,000 N, corresponding to a compressive strength of 550 MPa and a static strain of 0.055. Both tensile and compressive tests show that the fracture strength of <100>-oriented single-crystal silicon is 117 MPa at a strain rate of 0.001 s^−1^, but it increases to 550 MPa at a strain rate of 0.01 s^−1^.

## 5. Results

This study investigates the anisotropic dynamic mechanical behavior of single-crystal silicon at varying strain rates, employing nanoindentation and micro-tensile compression experiments. The key findings are summarized as follows:

(1) The indentation load–displacement curve demonstrates that phenomena such as pop-in, pop-out, and elbow can induce phase transformation in single-crystal silicon during testing. The elastic recovery rate of single-crystal silicon at room temperature is approximately 42%.
(2) The elastic modulus of single-crystal silicon demonstrates negligible size effects across varying strain rates. The average elastic moduli for the <100>, <111>, and <110> orientations are recorded as 163.34 GPa, 176.53 GPa, and 167.18 GPa, respectively. Conversely, hardness exhibits a positive correlation with the strain rate, with <111>-oriented silicon displaying notably higher hardness compared with its <100> and <110> counterparts.
(3) Room temperature fracture toughness of single-crystal silicon reveals substantial anisotropy; the <100> orientation exhibits the minimum toughness at 0.691 MPa·m^1/2^, whereas the <110> orientation shows the maximum toughness, 0.797 MPa·m^1/2^.
(4) Both tensile testing and compression testing indicate that single-crystal silicon undergoes fracture under a compressive load of 14,000 N, which translates into a compressive strength of 550 MPa and a static strain of 0.055. Specifically, <100>-oriented silicon exhibits a fracture strength of 117 MPa at a strain rate of 0.001 s^−1^, which escalates to 550 MPa when the strain rate increases to 0.01 s^−1^.

## Figures and Tables

**Table 1:** The analysis results of indentation tests on single-crystal silicon with varying crystal orientations, conducted at a consistent strain rate, are presented.

Strain Rate	Crystallographic Orientation	h_max_ (nm)	h_f_ (nm)	h_max_ − h_f_ (nm)	Elasticity of Response Rate
0.01 s^−1^	<100>	1252.58	671.25	581.33	46.41%
	<111>	1255.40	735.84	519.56	41.38%
	<110>	1266.17	724.17	542.00	42.81%
1 s^−1^	<100>	1275.43	738.33	537.10	42.10%
	<111>	1313.84	760.72	553.12	42.10%
	<110>	1279.76	748.22	531.54	41.53%

**Table 2:** Elastic modulus and hardness of <100> single-crystal silicon at different strain rates.

Crystallographic Orientation	E/H	0.01 s^−1^	0.05 s^−1^	0.10 s^−1^	0.25 s^−1^	0.50 s^−1^	1.00 s^−1^
<100>	E (GPa)	154.81 ± 1.94	167.93 ± 3.05	169.56 ± 2.53	169.48 ± 2.56	162.77 ± 3.98	155.48 ± 5.98
	H (GPa)	11.08 ± 0.12	12.66 ± 0.1 7	12.89 ± 0.21	13.29 ± 0.12	14.29 ± 0.34	15.31 ± 0.65
<111>	E (GPa)	172.13 ± 2.51	184.36 ± 4.11	184.17 ± 4.49	185.23 ± 5.94	167.89 ± 2.30	165.38 ± 0.40
	H (GPa)	11.27 ± 0.16	12.70 ± 0.16	12.76 ± 0.22	13.74 ± 0.12	14.68 ± 0.34	18.73 ± 0.00
<110>	E (GPa)	156.97 ± 3.02	156.97 ± 3.25	181.26 ± 3.70	176.25 ± 4.60	181.36 ± 4.27	150.25 ± 4.49
	H (GPa)	10.43 ± 0.24	12.17 ± 0.18	12.63 ± 0.27	13.24 ± 0.10	13.54 ± 0.16	17.04 ± 0.30

**Table 3:** Fracture length of single-crystal silicon in different crystal orientations under 300 mN load.

Crystallographic Orientation		1	2	3	4	5	Average Value
<100>	C (μm)	9.452	9.162	9.358	9.185	9.263	9.284
<110>	C (μm)	8.452	8.261	8.781	8.755	8.805	8.611
<111>	C (μm)	8.858	9.441	9.265	9.453	8.256	9.054

**Table 4:** Analysis of fracture toughness of single-crystal silicon with different crystal orientations under a load of 300 mN.

Crystallographic Orientation	δ	E (Gpa)	H (GPa)	F_max_ (mN)	C_a_ (μm)	K_IC_ (MPa·m^1/2^)
<100>	0.016	189.438	11.486	300	9.284	0.691
<110>	0.016	201.253	11.441	300	8.611	0.797
<111>	0.016	192.849	11.294	300	9.054	0.718

**Table 5:** Fracture toughness analysis results of Sample 1.

Strain Rate	0.01 s^−1^	0.05 s^−1^	0.10 s^−1^	0.25 s^−1^	0.50 s^−1^	1.00 s^−1^
α	0.016	0.016	0.016	0.016	0.016	0.016
E (Gpa)	165	168	171	168	168	177
H (GPa)	12.2	12.9	13.2	13.7	14.4	14.7
F_max_ (mN))	196.2	283.3	287.5	299.8	300.8	317.4
C1 (μm)	5.96	8.92	8.46	8.68	8.45	9.13
C2 (μm)	5.86	8.34	8.18	7.66	7.93	9.85
C3 (μm)	6.58	7.13	7.94	8.69	8.90	8.07
Ca (μm)	6.13	8.13	8.19	8.34	8.43	9.02
K_IC_ (MPa·m^1/2^)	0.761	0.706	0.706	0.697	0.672	0.650

**Table 6:** Fracture toughness analysis results of Sample 2.

Strain Rate	0.01 s^−1^	0.05 s^−1^	0.10 s^−1^	0.25 s^−1^	0.50 s^−1^	1.00 s^−1^
α	0.016	0.016	0.016	0.016	0.016	0.016
E (GPa)	165	168	171	168	168	177
H (GPa)	12.2	12.9	13.2	13.7	14.4	14.7
F_max_ (mN)	280.4	288.6	292.6	301.1	303.9	309.8
C1 (μm)	7.87	7.92	7.75	7.78	8.58	7.78
C2 (μm)	6.82	6.33	8.14	7.72	7.21	10.54
C3 (μm)	7.47	8.23	7.88	8.47	8.78	9.43
Ca (μm)	7.39	7.49	7.92	7.99	8.19	9.25
K_IC_ (MPa·m^1/2^)	0.821	0.813	0.756	0.747	0.709	0.611

**Table 7:** The <100> average fracture toughness of monocrystalline silicon at different strain rates.

Strain Rate	0.01 s^−1^	0.05 s^−1^	0.10 s^−1^	0.25 s^−1^	0.50 s^−1^	1.00 s^−1^
F_max_ (mN)	280.4	288.6	292.6	301.1	303.9	309.8
K_IC_ (MPa·m^1/2^)	0.791	0.760	0.731	0.722	0.691	0.631

## Data Availability

Data are contained within the paper.
